# Building resilience in oncology teams: Protocol for a realist evaluation of multiple cases

**DOI:** 10.1371/journal.pone.0268393

**Published:** 2022-05-12

**Authors:** Dominique Tremblay, Nassera Touati, Kelley Kilpatrick, Marie-José Durand, Annie Turcotte, Catherine Prady, Thomas G. Poder, Patrick O. Richard, Sara Soldera, Djamal Berbiche, Mélissa Généreux, Mathieu Roy, Brigitte Laflamme, Sylvie Lessard, Marjolaine Landry, Émilie Giordano

**Affiliations:** 1 Faculty of Medicine and Health Sciences, Université de Sherbrooke, Longueuil, Québec, Canada; 2 Centre de Recherche Charles-Le Moyne, Longueuil, Québec, Canada; 3 École Nationale d’administration Publique, Montréal, Québec, Canada; 4 Faculty of Medicine and Health Sciences, McGill University, Montréal, Québec, Canada; 5 Susan E. French Chair in Nursing Research and Innovative Practice, Montréal, Québec, Canada; 6 Department of Management, Evaluation and Health Policy, School of Public Health, Université de Montréal, Montréal, Québec, Canada; 7 Centre de Recherche de l’Institut Universitaire en Santé Mentale de Montréal, Centre Intégré Universitaire de Santé et de Services Sociaux de l’Est-de-l’Île-de-Montréal, Montréal, Québec, Canada; 8 Institut National de Santé Publique du Québec, Montréal, Québec, Canada; 9 Maison Michel-Sarrazin, Québec, Québec, Canada; 10 Department of Nursing, Université du Québec à Trois-Rivières, Trois-Rivières, Québec, Canada; 11 Centre de Recherche du CHUS, Sherbrooke, Québec, Canada; Public Library of Science, UNITED KINGDOM

## Abstract

**Background:**

Teams caring for people living with cancer face many difficult clinical situations that are compounded by the pandemic and can have serious consequences on professional and personal life. This study aims to better understand how a multi-component intervention builds resilience in oncology teams. The intervention is based on a salutogenic approach, theories and empirical research on team resilience at work. This intervention research involves partnership between researchers and stakeholders in defining situations of adversity and solutions appropriate to context.

**Methods:**

The principles of realist evaluation are used to develop context-mechanism-outcome configurations of a multi-component intervention developed by researchers and field partners concerned with the resilience of oncology teams. The multiple case study involves oncology teams in natural contexts in four healthcare establishments in Québec (Canada). Qualitative and quantitative methods are employed. Qualitative data from individual interviews, group interviews and observation are analyzed using thematic content analysis. Quantitative data are collected through validated questionnaires measuring team resilience at work and its effect on teaming processes and cost-effectiveness. Integration of these data enables the elucidation of associations between intervention, context, mechanism and outcome.

**Discussion:**

The study will provide original data on contextual factors and mechanisms that promote team resilience in oncology settings. It suggests courses of action to better manage difficult situations that arise in a specialized care sector, minimize their negative effects and learn from them, during and after the waves of the pandemic. The mechanisms for problem resolution and arriving at realistic solutions to professional workforce and team effectiveness challenges can help improve practices in other settings.

## Introduction

Professional oncology teams are exposed to many situations of adversity [1–4]. Adversity refers to events that are stressful and likely to have a negative effect on health or well-being. The risk to clinicians of mental and physical health problems has been heightened by the COVID-19 pandemic [5]. An international study of 1,520 oncology clinicians shows that one quarter are at risk of distress, over a third feel emotionally drained, and two thirds state they can no longer do their work well. Resilience, defined as the capacity to face and recover from situations of adversity, is also identified as one of the main predictors of well-being among clinicians [6]. These results highlight the importance of interventions to support resilience, but also suggest that improving the health and well-being of individual clinicians can help assure the maintenance of oncology services. However, given that teamwork is vital to caring for people living with cancer, resilience must go beyond the individual. Situations of adversity can compromise team functioning [7]. As well, team resilience is more than the sum of the resilience of individual team members [8] and tailored team-level supports are needed. The pandemic context makes it more important than ever to develop and evaluate interventions for building team resilience and produce relevant knowledge that can be used rapidly to minimize the repercussions of adverse situations, including those related to COVID-19 [9].

Oncology practice involves numerous adversity situations. For example, care teams must deal with informing patients of a serious diagnosis, delivering bad news about disease progression, and keeping up with the multitude of new treatments, clinical trials [10, 11], constantly evolving knowledge [12], treatment specifications for different age groups [13], and the rise of controversial therapies [14]. At the organizational level, care provided over a long period of time by multiple clinicians working in different settings generates challenges of communication and coordination [15, 16]. These specialized teams, juggling with increased demand and a scarcity of resources, are subject to high staff turnover and absenteeism [17, 18]. Several studies show that the demanding work of cancer care heightens the risk of deterioration in the well-being, health and functioning of oncology teams [1, 3, 4, 19, 20]. Adversity related to COVID-19 adds to daily stressors: managing personal protection, confronting service rationalization or reductions [21], adapting triage processes in oncology [22], using telemedicine and increasing delays in diagnosis [23]. The accumulation of these adverse conditions presents a threat to team functioning, while interventions to build resilience in specialized care teams remain scarce. Scholars agree that adversity is an essential condition to observe resilience behaviour [24]. The nature, frequency and intensity of adversity varies, but these situations share the potential to bring about dysfunction and, in the end, compromise the achievement of team objectives [25]. The context of oncology teams therefore offers a typical case to better understand team resilience at work, explore promising interventions and, in the end, help maintain access to specialized care in times of adversity.

This multiple case study protocol using principles of realist evaluation proposes a multi-component intervention for oncology teams in Québec (Canada). It has potential for significant impact as it: 1) recognizes that teamwork is critical to caring for people living with cancer [12, 26]; 2) addresses priorities of the Canadian Strategy for Cancer Control (2019–2029), namely 2a) providing high quality care in a sustainable health system; 2b) overcoming barriers that prevent people from obtaining the care they need [27]; 3) aligns with the *Quadruple Aim* framework recognized internationally for contributing to quality care, notably by improving the well-being of providers [28]; and 4) is embedded in an interventional research project [29]. The approach reaches and involves multiple collaborators with concrete experience of adversity and potential solutions, while also increasing knowledge [30].

The study aims to better understand how a multi-component intervention builds resilience in oncology teams in Québec (Canada). More specifically, its objectives are to: 1) describe the contextual factors (individual, team, organizational) most important in enabling and impeding team resilience at work in oncology; 2) analyze how, why, for whom and under what circumstances the target mechanisms of the intervention act on team resilience at work in oncology; 3a) measure the intervention’s outcomes on team resilience at work in oncology, health-related quality of life and team functioning; and 3b) explore the cost-effectiveness of the intervention.

### The Building Resilience in Oncology Teams (BRIOT) multi-component intervention

#### Conceptual background

Resilience has been studied primarily at the individual level [31, 32]. Only recently has team resilience received attention in theoretical and empirical studies [7, 33–36]. While conceptual ambiguities persist, team resilience is defined as a dynamic process to effectively face adversity experienced by all members of a team. Three main mechanisms are mobilized: minimizing, managing and mending. Minimizing involves monitoring and preparation to minimize the impact of adversity; managing involves management and adaptation during a situation; and mending involves restoring balance and learning for the future [24, 34].

Resilience is recognized as an essential characteristic among healthcare professionals [37], enabling them to maintain meaning at work during times of adversity [38]. However, apart from two studies undertaken in large Canadian urban centres, namely Toronto [39] and Greater Montreal [40], empirical research on resilience in oncology teams remains rare. This is partly due to the relatively recent focus on the concept of team resilience, the lack of consensus on a conceptual framework, and the poor consistency of empirical research with clinicians due to a lack of specific measurement tools [37]. Our study contributes to the effort to fill gaps in empirical research on team resilience [41], looking specifically at healthcare professionals, including doctors [38].

#### Theoretical foundations

Three complementary theoretical perspectives inform the conceptual framework that guides the present study.

The first is team resilience at work “*R@W Team*” [8, 42], which assembles the essential elements of team resilience operating in an environment marked by uncertainty, complexity and pressure (as seen in oncology). Team resilience at work is dynamic and can be developed by emphasizing strengths instead of weaknesses. For this reason, our multi-component intervention focuses on reflexive activities that lead teams to identify their strengths and enlist them to promote well-being. The ‘Team’ component of R@W serves to guide and support the intervention and operationalize seven dimensions of team resilience [8, 43]: resourcefulness, robustness, perseverance, self-care, capability, connectedness and alignment. This perspective is supported by empirical studies showing a positive association between team resilience and team functioning [24, 44], team cohesion [36], and team member health and well-being [1–4, 19, 20, 39].

The second perspective draws on the salutogenic theory (i.e., how health is produced or maintained despite adverse situations), which positions the intervention within health promotion efforts [45]. It emphasizes the identification of resources, conditions and factors that allow to face difficult situations and to promote health. The Building Resilience in Oncology Teams (BRIOT) intervention is complementary to individual-level therapeutic measures applied once a health problem is manifest, and aims to prevent and minimize the negative effects of situations of adversity, manage them collectively and "learn about and through practice" [46]. This salutogenic approach promotes a sense of coherence when facing stressful situations [45, 47], because "sense-making" protects against anxiety, depression and burnout, and predisposes to individual and collective resilience [48].

**Fig 1:**
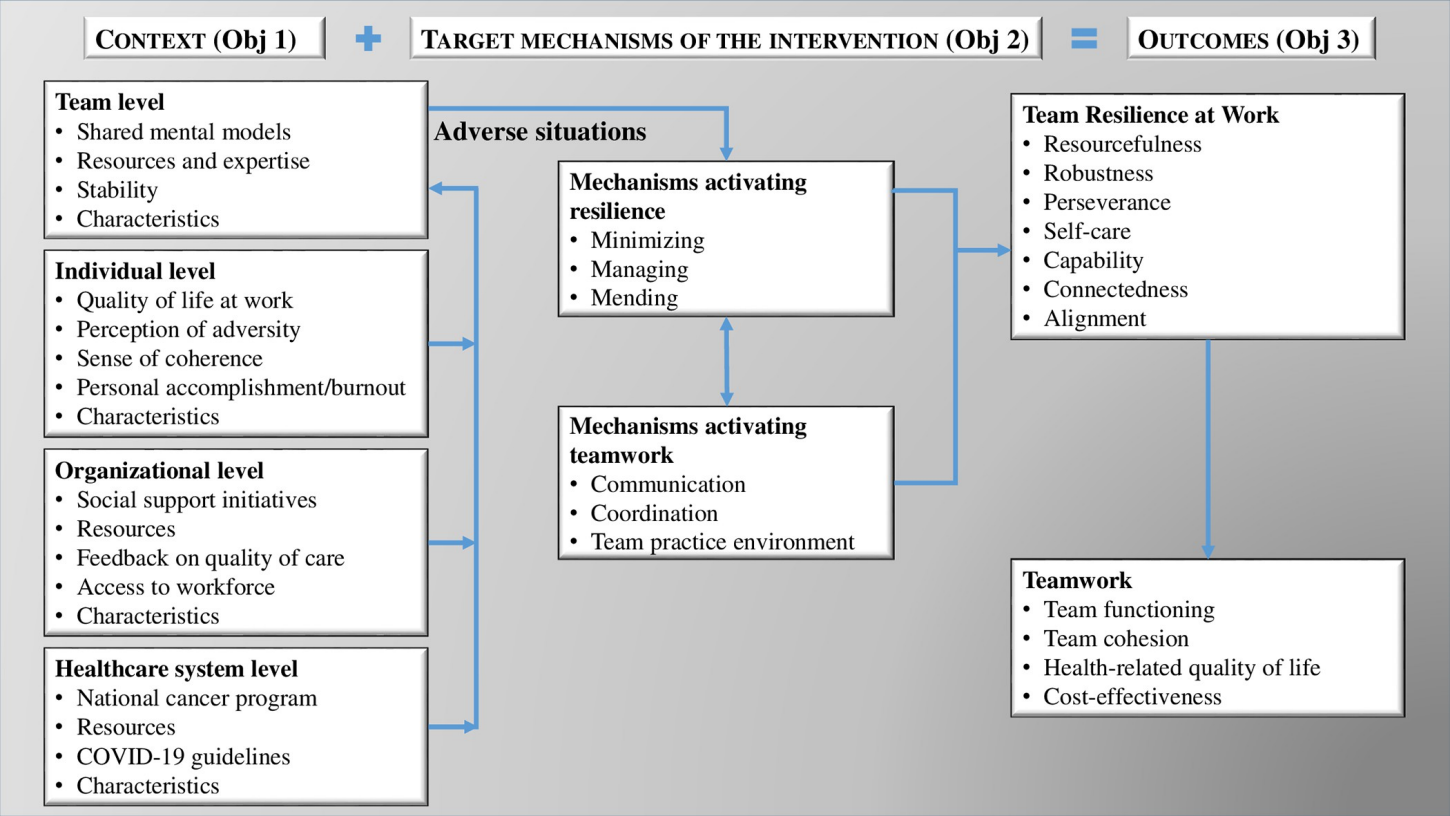
Conceptual framework of team resilience at work in oncology.

The third perspective rests on theories of teamwork, where mechanisms of communication, coordination and creation of a pleasant environment are mediators of team resilience associated with functioning, cohesion, and health [26, 49]. The operationalization of these perspectives takes into account the multi-level context in which care teams evolve (i.e., team, organization, healthcare system). Fig 1 synthesizes the contextual factors, mechanisms and anticipated outcomes of the BRIOT intervention.

#### Operational aspects

The BRIOT intervention is participatory and integrates the expertise of teams to confront situations of adversity by mobilizing their problem-solving capacities [34]. A multi-component intervention is suited to complex, evolutive situations that depend strongly on context [50], such as team resilience at work. This type of intervention has a core based on research evidence while allowing the periphery to adapt to local context [51]. A planning stage to prepare field work involves: 1) assessing the local state of affairs (organizational characteristics and administrative data); 2) introducing the project to teams (presentation of the study, mutual engagement between researchers and participants, identification of local champions and team representatives); 3) observing clinical team meetings; 4) administering a baseline questionnaire (as described in the next “Variables” section) for the team to complete pre-intervention.

The BRIOT intervention is built around four components that are broken down into team-based reflexive activities (Table 1). Globally, the components aim to identify situations of adversity and characterize the activation of mechanisms that promote resilience: monitoring and preparation to minimize the negative effects of situations of adversity, managing such situations when they arise, and learning for the future; and mechanisms that promote teamwork: communication, coordination, creation of a positive team practice environment. Collective reflexion contributes to understanding and making sense of adversity, notably in the context of COVID-19 [52]. Sequential components 1, 2 and 4 (described in Table 1) are undertaken in 90 minutes every 4 weeks, at a time chosen by the team. Working from participant perceptions, the narrative activities aim to characterize the nature and extent of the adverse experience, and identify resources and concrete actions that help team members face it together. Component 3 completes the narratives with a questionnaire to measure certain contextual factors, mechanisms and the outcomes of the intervention on team resilience. Results at each time point are interpreted with the local team in order to identify action targets for optimizing mechanisms that promote team resilience. A debrief (with designated champions, managers, patient representatives) will be undertaken following each component to prioritize actions that can promote facilitators, overcome obstacles and maintain the objectives of care provision. These findings are assembled to co-construct, with the local team, the logic model of the intervention [53]. Components of the BRIOT intervention are led by the researchers, with participation of a knowledge broker [54] and note-taker to record team dynamics.

**Table 1:** The BRIOT multi-component intervention.

COMPONENTS (C)	ACTIVITIES
**Target mechanism: Monitoring and preparation for situations of adversity (Minimizing)**	
C^1^- Reflexive discussion (vignette) [55] around a plausible situation of adversity: new diagnosis or progression of cancer. Video excerpts from: *Les messagers de l’impossible* [56]	• Nature and magnitude of situations to be monitored • Predictability and severity of adverse situations • Examples of situations where the team has shown robustness/stress • Resources available in stressful situations • Examples of perseverance/problem-solving
*Debrief*: *Strategies for monitoring*, *informing and alerting*	
**Target mechanism: Managing the response to situations of adversity (Managing)**	
C^2^—Reflexive discussion around Photos (photovoice) [57] on managing adversity	• Maintaining the ability to achieve common goals • Developing shared mental models and maintaining alignment in the face of adversity • Strategies for taking care of each other • Optimizing internal team resources • Optimizing external team resources • Designating mentors and clarifying their role in the team
*Debrief*: *Team mobilization in response to adversity*	
**Target mechanism: Communication, coordination, team practice environment**	
C^3^—Questionnaire: • Quality of life at work [58] • Impact of adversity caused by COVID-19 • Sense of coherence [59] • Personal accomplishment/burnout [60] • Sociodemographic characteristics • Teamwork mechanisms [61–64] • R@W Team [8] • Team functioning [65] • Team cohesion [66] • Health-related quality of life [67, 68]	• Measurement and interpretation of results • Reinforcement of positive items • Prioritization of actions to address less positive items
*Debrief*: *Identification of teamwork (antecedents*, *mechanisms*, *outcomes) relative to resilience*	
**Target mechanism: Recovering and Learning from the experience (Mending)**	
C^4^—Reflexive discussion on the intervention process, uptake and sustainability of BRIOT [69]	• Feedback on integrated results of group narratives and questionnaires • Tools to monitor adverse situations and recovery strategies • Principles of wellness-promoting actions • Logic model to guide continuity
*Debrief*: *Summary*, *future activities*, *responsibilities/problems-solutions-resources*	

## Materials and methods

### Research approach

The study draws on the principles of realist evaluation [70] to achieve its objectives. These principles serve to specify Context-Mechanism-Outcome (C+M = O) associations to explain how, why, for whom, with whom and under what conditions the intervention is undertaken and produces (or does not produce) the anticipated outcomes [70–72]. We have used this type of evaluation in prior studies [73–75]. Realist evaluation is appropriate in interventional research where problems and useful and appropriate solutions are arrived at through dynamic exchanges between knowledge producers and users [76, 77], or during reflexive discussion. The context (Obj1) considers critical factors in individual, interpersonal, institutional and infrastructural transformation, described by Pawson and Tilley as the "Four I’s" [70]. Mechanisms (Obj2) refer to components, processes or structures that are the ultimate causes of the emergence of outcomes anticipated from an intervention in a given context [78, 79]. Mechanisms are largely dependent on the reasoning and willingness of actors, which justifies the use of reflexive team activities as a component of the intervention. Mechanisms are activated when a team undertakes actions and assembles conditions that will help achieve the expected outcomes. According to Dalkin et al. (2015), mechanisms are observable in team actions to transform their practices in a given context [79]. Mechanisms are characterized based on the integrated narrative and questionnaires completed pre-intervention (T_0_) and during the intervention (T_1_). Outcomes (Obj3) reflect the achievement (or not) of anticipated outcomes on the main variable, namely team resilience at work in oncology, and secondary variables (team functioning, team cohesion, health-related quality of life, cost-effectiveness).

### Study design

The multiple case study design [80] is coherent with realist evaluation [71]. This design is well suited to explaining contemporary collective action (multiple actions by multiple actors) phenomena (BRIOT) in a natural setting (oncology teams) when it is impossible to control the numerous variables [80]. The multiple case study offers a representation of the population of oncology teams in their natural environment, which can influence their perception of an intervention, its mechanisms of action, and its outcomes [81]. The case study relies on multiple sources of data in order to deeply explore a phenomenon with a large number of variables and a limited number of cases [80]. Qualitative and quantitative data are mobilized according to convergent mixed methods embedded in complementary fashion [82]. Intra-case analysis helps understand how context+mechanism associations produce outcomes. Inter-case analysis enhances understanding of recursive models that explain how and why outcomes converge or differ depending on their context and the activated mechanisms [83].

### Setting

The setting for each case is an Integrated Health and Social Service Centre (IHSSC or IUHSSC when it includes a university centre) that offers oncology care and services. The cases are instrumental and contribute to the explanatory power of the study by revealing the meaning actors attach to the intervention in their daily practice context [81]. Four cases are chosen for their potential to elucidate the activation of mechanisms and various context characteristics (geographic location, population served, university mission, size and composition of oncology teams). The selected cases allow us to appreciate how the intervention is adapted in natural settings in order to target actions that would help teams face adversity. The unit of analysis is the interdisciplinary team made up by clinicians and surrounded by managers and directors involved directly or indirectly in the care of people living with cancer.

### Ethics approval and consent to participate

The research project was approved by the Research Ethics Board of the Centre intégré de santé et de services sociaux de la Montérégie-Centre, which serves as the review board for the other sites in this multicenter project (Project number MP-04-2022-675). All participants will sign an informed consent form in accordance with the ethical policy for research involving humans [84] and the standards of participating sites.

### Qualitative approach

The qualitative approach aims to characterize the most important context factors (Obj1) and mechanisms (Obj2) that could potentially be activated or enhanced by the intervention. It relies on interpretive description [85] where criteria for reporting qualitative research are applied [86].

#### Qualitative data collection

Qualitative data will be collected from four sources using different methods [80] at strategic moments of the study: 1) document review (e.g., meeting minutes, action plans, local administrative reports, government documents, scientific papers) at the outset of the study to enable us to describe the state of affairs and specify a number of interview questions; 2) observation (e.g., meetings of administrators or interdisciplinary teams) at the start of the study to gain an idea of the teamwork mechanisms already activated or in need of enhancement (e.g., communication, coordination, team practice environment); 3) group interviews [87] held around the reflexive activities and debriefs described in Table 1; 4) individual semi-directed interviews with key informants at various levels following the intervention to validate findings and the logic model of the intervention (e.g., Cancer Directorate at Ministry level, co-managers of the cancer program, members of the administration and people living with cancer at each site) [88]. About 15 interviews per case and another 10 at system level (n total = 70) are expected, with the final number adjusted according to the richness and redundancy of data [89, 90]. Guides for individual and group interviews are adapted to the type of actor (S1 File) and based on elements of our conceptual framework (Fig 1 and S2 File). All interviews will be audio recorded and transcribed, and data from all sources will be stored in a formal database managed with QDA Miner 5 software [91]. The triangulation of sources offers a deep perspective and contributes to the internal validity of qualitative components [92–94].

#### Qualitative data analysis

Data will be analysed in an iterative process of data condensation [95]. Cycles of content analysis structure data in order to develop logic patterns through: 1) descriptive analysis to arrive at first level concepts via structural coding according to our framework (Fig 1); 2) second level thematic categorisation and quantification through interpretive analysis in which concepts are transformed into critical themes/variables according to their presence or absence; 3) contrasting analysis to arrive at third level aggregate dimensions through the development of synthetic tables to establish links between factors and identify the mechanism at work. Results in each cycle will be discussed among researchers and collaborators in the field to ensure that findings reflect user perspectives, in line with the interventional research approach. Participant characteristics, including gender, will be considered in the process of data condensation [96]. Intra-case analysis, followed by inter-case analysis [81] will reveal differences and similarities in recursive models (reproducible semi-regularities) [70] with strong potential to explain how, why, with whom, for whom and in what context the intervention acts on the main variable (team resilience) and secondary variables (team functioning, team cohesion, health-related quality of life, cost-effectiveness) [97].

### Quantitative approach

The quantitative approach mobilizes a transversal study design [98] to enable the exploration of Context+Mechanisms = Outcomes associations when a randomized control trial is not optimal to understand a complex phenomenon in a natural setting [99]. This part of the study complements the qualitative exploration (S2 File).

#### Quantitative data collection

Quantitative data will be collected through validated French language questionnaires with a completion time as short as possible. Data will be collected at baseline (T_0_) and following components 1 and 2 (T_1_). The T_1_ questionnaire is completed after reflexive team discussion of results and will identify the gains, challenges and actions that can overcome barriers to resilience. A brief survey will be conducted after each component regarding the participants’ appreciation of group discussions, perceived quality of life at work and perceived impact of adversity related COVID-19.

#### Variables

The main dependent variable is resilience at work in oncology teams. It is operationalized with one of the rare tools dealing with team resilience at work, the French version of the *R@W Team Scale* (42 items; α = 0.95), which measures seven dimensions of team resilience: resourcefulness, robustness, perseverance, self-care, capability, connectedness, alignment [8]. While there are other tools to measure resilience, none is specific to health professionals [37]. Secondary dependent variables are: *team functioning*, measured using the Rousseau scale (12 items: α = 0,84) [65], which has been used with oncology teams in our prior work [100]; *team cohesion*, measured by the dimension Team Cohesion (7 items; α = 0,86) of the *Interdisciplinary Team Performance* instrument [66]; *health-related quality of life* of team members, assessed using the *CORE-6D*, which includes a Rasch analysis and a temporal arbitration method to elicit utility values with excellent predictive value (R^2^ = 0.99). The CORE-6D includes five mental health dimensions and one physical health dimension on a Likert-type scale [67]. To ensure the robustness of results, the SF-6Dv2 will also be used as a secondary measure [68, 101]. Differences in results on the CORE-6D at different time points will enable us to determine changes in quality of life (*Quality-Adjusted Life Years (*QALY)) [102]. QALYs *cost-effectiveness* enable evaluation of interventions in relation to costs. These data, at the initial stage of the intervention, are crucial for resource allocation and decision-making [103].

The variables linked to context in our conceptual framework are the following. The *quality of life at work* (QLW) is assessed with the QLW Thermometer (scale 0–100): (0–25:red = problem zone; 26–50:yellow = needs improvement zone; 51–100:green = good QLW zone) [58]. The perceived *impact of adversity caused by COVID-19* is measured on a visual analog scale (0 = no impact; 100 = significant impact). The *sense of coherence*, the core concept of salutogenesis, measures positive adaptation to stress to remain healthy [47]. It is measured with the three dimensions (i.e., comprehensibility, manageability and meaningfulness) of the multidimensional construct of the *Work-SoC-9* (9 items; α = 0.83) [59] according to a 7-point differential semantic scale ranging from positive to negative perception (e.g., controllable–uncontrollable). Two of the co-authors (M Généreux, M Roy) use the SoC in their studies [52, 104]. The *personal accomplishment/burnout* is measured with the French-language version of the Maslach Burnout Inventory (MBI) [60] (8 items; α = 0.72) on a 7-point scale.

Variables related to team mechanisms are measured as follows. Team *communication and coordination* are measured with the *Relational coordination* (7 items; α = 0.86) 5-point scale [61–63]. Communication and coordination are positive and significant predictors of team cohesion [66]. To measure *team practice environment*, we use the short version of the *Practice Environment Checklist mini-PEC* (5 items, α = 0.82) [64]. Mechanisms to face adversity–*minimizing*, *managing*, *mending* [24]–are integrated by quantifying qualitative data, attributing a score (present = 1; absent = 0) to third level aggregate dimensions from qualitative analysis [105].

The following characteristics will be used as control variables. *Sociodemographic and professional characteristics* include age, gender, gender-related roles, education level, type of profession, professional experience, professional experience in the oncology team, work status, role. Gender-related role is determined using the *Labour Force Gender Index (LFGI)* [106], with 4 dimensions dealing with social role rather than biological sex. *Team characteristics* refer to team size and team composition, which includes types of professions and diversity of team members, and mean team characteristics (average age, professional experience, professional experience in the team) [66]. *Organizational characteristics* are based on information from the year preceding the pandemic (March 2019-March 2020), and in the 12 months before and after the start of the intervention (e.g., mandate, academic affiliation, size of deserved population, geographic location, staff turnover). Other organizational elements focus on social support initiatives, resources, feedback on quality of care, access to workforce. *Healthcare system characteristics* refer to information regarding the National cancer program, resources, and COVID-19 guidelines.

The self-administered digital questionnaire takes about 30 minutes to complete. Five minutes are also reserved at the end of each group interview to report on participant’s appreciation (3 items) [107], the perceived quality of life at work (thermometer) [58] and the perceived impact of adversity caused by COVID-19 (visual analog scale).

#### Population, sample, and sample size

The reference population is made up by oncology teams in Québec. The convenience sample is composed of natural oncology teams in Québec [108]. Inclusion criteria are: being a member of an oncology team for more than a year; having been at work during the 12 months preceding the intervention; being willing to participate in all the activities of the intervention.

The sample size according to information from the study sites is a total of 210 participants, considering a global minimal response rate of 35%. Calculation of size and power is done on the main result of the intervention, namely the team resilience at work score, for which there is no pre-established cut-off. With 210 participants in a multivariate linear regression, the resilience score where the coefficient of determination R^2^ global with 20 predictors would be 0.85, and a reduced R^2^ with 10 predictors would be 0.80 with a risk α of 0.05, giving a power of 1-β > 0.99.

#### Quantitative data analysis

Analyses will be conducted with SPSS (version 24) [109] and SAS (version 9.4) [110] software. Results with p < 0.05 are considered significant. Descriptive statistics per item and sub-scale are used to summarize the variables. Analyses stratified by gender for the Gender Index are explored (given the high proportion of female nurses) to determine if gender is associated with the perception of resilience and the outcomes of the intervention. To identify differences between T_0_ and T_1_, Student or non-parametric tests such as Mann-Whitney and ANOVA are used with continuous variables. For categorical variables, Chi square tests are used. Univariate and multivariate linear and logistic regression analyses are conducted to explore the link between team resilience and the other variables, while controlling for potentially confounding variables (individual and organizational characteristics). The need to adjust p-values will be assessed to compensate the possibility of increasing Type 1 errors when multiple measures are used. The Bonferroni correction is the classic method used in statistics to correct the threshold of significance in multiple comparisons. This method is considered excessively conservative. To the best of our knowledge, while alternate methods exist, there is no consensus on a gold standard [111].

Cost-effectiveness estimates are made from the perspective of the healthcare network. Methodologies follow recommendations of the Canadian Agency for Drugs and Technologies in Health [112]. A deterministic approach is used, given the number of teams involved. An annual discount rate of 1.5% is employed to update costs and effects. First, the cost of resources used in the intervention are recorded using a scorecard, including personnel costs and consumables. The different variables associated with outcomes of the intervention are then used differentially to establish an incremental cost-effectiveness ratio, with particular attention to the CORE-6D that allows for calculation of cost per QALY.

### Quality criteria for the study

The credibility of the qualitative approach rests on a synthesis of solid theoretical foundations [95]. Reliability comes from the triangulation of data sources, the methods and the researcher-collaborator partnership, along with the maintenance of a journal to record each step of the process [95]. Transferability is enhanced through rich description of context factors and mechanisms [95]. Internal validity of the quantitative analysis is assured through the use of questionnaires with established psychometric validity in the population under study (healthcare professionals), uniformity in procedures, and analysis by research team members with solid experience in statistics (D Berbiche, TG Poder). External validity rests on the generalizability of results. The integration of complementary qualitative and quantitative data helps compensate recognized limits of each approach [82].

### Integrated knowledge translation

This interventional research is conducted in close partnership with knowledge users (Table 2) and includes both integrated knowledge translation and end-of-project approaches [113]. Our team places considerable importance on bidirectional exchanges between producers and potential users of research, with knowledge users involved throughout the process. This approach is strategic as it can have a positive influence on the adoption of new practices based on research findings, and can contribute to solving complex problems [72, 114]. Integrated knowledge translation is already underway in the pilot study and preparation of this protocol. The components of the *Knowledge to Practice* framework are the cornerstone of our integrated knowledge translation plan [115].

**Table 2:** Integrated and end-of-project knowledge translation activities.

Target	Activities
On-site collaborators: clinicians, managers, policymakers, people living with cancer	• Early identification of barriers, facilitators, controversies, and potential solutions • Access to the research team in the field • Discussion of interim findings at each site • Debriefing meetings to prioritize actions and design the follow-up plan • Newsletter published every 4 months on our website, on the BRIOT Collaborative Space accessible to all, on co-researcher and collaborating partner networks • Support from co-researchers to designated local champions and managers • End-of-project report at each site
Dissemination: academic and research communities	• Engage the next generation of oncology researchers and assure the future of the cancer care system: graduate students, physicians and professionals • Dissemination of the study and its results in courses taught by co-investigators in their respective universities • Mobilize research networks (e.g., RRISIQ, Réseau-1 Québec, Unité de soutien SSA Québec) • Share tools developed and deemed useful for team building and professional well-being
Dissemination: large scale, marketing on social media, knowledge broker	• Mobilize partner networks: MSSS Cancer Program, Collaborative Space, Local and Regional Cancer Committees of IHSSCs and IUHSSCs • Use university and inter-university networks: e.g., RRISIQ, Research Chair on improving the effectiveness of care for people living with and beyond cancer, Communities of Practice in Cancer Care • Scientific Conferences: e.g., MASCC, Canada’s Applied Research in Cancer Control Conference, UICC, ASCO Palliative and Supportive Care in Oncology Symposium • Website related to our work: http://cancerinnovation.ca/ and professional social networks • Publications: e.g., Implementation Science, Supportive Care in Cancer, BMC Health Services Research
Targeted dissemination	• Adapt synthesized content for decision-makers, clinicians and the general public

Abbreviations: ASCO, American Society of Clinical Oncology; BRIOT, Building Resilience in Oncology Teams; IHSSC, Integrated Health and Social Service Centre; IUHSSC, Integrated University Health and Social Service Centre; MASCC, Multinational Association of Supportive Care in Cancer; MSSS, Ministère de la Santé et des Services sociaux; RRISIQ, Réseau de recherche en interventions en sciences infirmières du Québec/Quebec Network on Nursing Intervention Research; SSA, Système de Santé Apprenant/Learning Health System; UICC: Union for International Cancer Control.

## Discussion

Our approach takes its inspiration from components of the UK National Health Service’s model [116], which offers a means of identifying strengths and weaknesses in the implementation of improvement initiatives and predicting the likelihood of their sustainability. This UK model includes three main dimensions [116] with specific factors that would help assure the sustainability of the BRIOT intervention: 1) the intervention process (perceived benefits, adaptability, monitoring of progress); 2) intervenors (involvement, behaviour, support for legitimate leaders, organizational support); 3) organizations (alignment with values and culture, support infrastructure). Monitoring of these factors can be recorded and serve to analyze situations of adversity and solutions (minimizing, managing, mending) [117]. This approach will require conditional measures (*step-wise approach*) [118] that can be adapted to address unforeseen developments that may compromise sustainability.

We anticipate a number of challenges in the proposed multi-component intervention and consider the following means of addressing them. Our expertise and strategies will help advance the research while adapting to challenges arising in the field [119]. The main foreseeable challenges (*in italics*) and ways to address them are: 1) *participation of local teams*: information sessions on the expected benefits of the study and components of the intervention to generate interest among various participants; constitution of a local committee including "champions" and clinical-administrative co-managers for debriefing sessions and to assure follow-up according to a step-wise problem resolution approach [118]; support from directors and managers to facilitate participation; monetary compensation for participation; familiarity with medical and professional culture and with accompanying teamwork [120]; 2) *time required to participate*: timing of intervention activities and modalities for individual and group interviews; selection of questionnaires that are as short as possible, given the already heavy workload of oncology teams; advantages of speaking about their experience; 3) *fit between the research team (designated researcher per site) and collaborators*, *with clear roles*: establishment of a Steering Committee (coordination and monitoring) and an Advisory Committee (interpretation of results, planning the stages of the intervention) and systematic evaluation of the conduct of meetings [107] to signal the need for action to sustain interest; 4) *management of large quantities of data* [121]: computer storage and management on QDA Miner (access controlled and limited to the research team and research professionals) to enable the integration of qualitative and quantitative data and create tables and figures helpful to framing the intervention [69]; 5) *complementarity* with other team consolidation efforts to assure the rapid dissemination of findings and avoid duplication of interventions.

## Conclusions

Interventional research approaches, where co-production is emphasized, lead oncology teams to pay close attention to their members in order to generate health. This approach and our interdisciplinary perspective will enable us to: 1) better understand how team resilience mechanisms are put in place as the intervention matures, and how these mechanisms contribute to developing capacities for resilience; 2) produce new knowledge on best practices for building resilience, and on the impact of team resilience during and after the waves of COVID-19; 3) find out whether gender influences the implementation and/or perceived outcomes of the intervention; 4) support decision-making at various levels of governance, where there is considerable pressure to improve care for people living with cancer while also better managing scarce resources [17]; 5) contribute to filling gaps in empirical research on team resilience at work in the health sector; 6) provide cost-effectiveness data to support decision-making around activities associated with teamwork, which are now poorly documented; 7) produce detailed data that supports the transferability of findings to other teams caring for people living with cancer (primary care, palliative care) or other groups living with chronic diseases.

## Supporting information

S1 File Interview guides for qualitative data collection. (DOCX)

S2 File Elements evaluated in the study. (DOCX)
