# Supplementary material for: Building resilience in oncology teams: Protocol for a realist evaluation of multiple cases
Source: PLoS One. 2022 May 12;17(5):e0268393. doi: 10.1371/journal.pone.0268393 (PMC9098052; doi:10.1371/journal.pone.0268393)
Supplement: S1 File — (DOCX) [file pone.0268393.s001.docx]

**S1 File. Interview guides for qualitative data collection**

This additional file includes the following interview guides: A) Group interview guide for component C^1^: Vignette; B) Group interview guide for component C^2^: Photovoice; C) Group interview guide for component C^4^: Uptake and feedback; D) Individual interview guide for key informants.

1. **Group interview guide for component C^1^: Vignette**

| **Participating site ID** |  |
| --- | --- |
| **Group ID code** |  |
| **Date** |  |
| **Start time** |  |
| **End time** |  |
| **Interviewing team members and roles** | Interviewer:  Co-interviewer:  Note-taker: |
| **Introduction and ground rules** | |
| **Interviewer’s introduction** | Thank you for taking the time and making yourselves available for this group discussion and for the project. |
| **Confidentiality and anonymity** | I would like to remind you that any information you reveal here will remain confidential. Under no circumstances, your names or occupations will be transmitted when the results of the study will be presented or published. If names are mentioned during the discussion, they will be anonymized. Only members of the research team will have access to the information for analysis purposes, and they are all committed to respecting the confidentiality of information.  You can withdraw from the study at any time if you want to, without any consequences. Do you have any questions?  Please read and sign the consent form. |
| **Audio recording** | This interview will be audio recorded. The audio recording will be transcribed to respect anonymity.  Please feel free to ask me to stop the recording at any moment for specific questions if needed.  Also, if you wish to remove some content, you can ask me during or immediately after the interview. |
| **Introduction** | I will ask you some questions about the different aspects of resilience at work in oncology teams. What I am interested in is your perspective, experience and perceptions on the subject, so feel free to add anything you think is relevant: there is no good or bad answer or point of view. |
| **Themes** | **Questions for collecting qualitative data** |
| **Shared mental models**  **(Resilience at work)** | 1. When we talk about “Team resilience at work in oncology”, what does it mean to you?    1. Positive discourse?    2. Negative discourse?    3. Outcomes on teamwork? |
| **Watch video extract 1 of the vignette** | |
| **Resources and expertise**  **(Communication, Coordination, Team practice environment)** | 1. A. What resources and expertise do you have within your team that help you face difficult situations? 2. Expertise/competence 3. Human capital 4. Back-up from colleagues 5. Lessons from previous situations 6. Mutual trust   B. What resources and expertise are available outside your team that help you face difficult situations?   - 1. Norms and standards of practice   2. Social support initiatives   3. Continuing education   4. Employee assistance programs   5. National Cancer Program   6. National Cancer Plan |
| **Resources and expertise (Team functioning, Team cohesion, Health-related quality of life)** | 1. How is leadership expressed within your team to help you recover from a difficult situation?   a. Leadership: includes functional leadership and collective leadership around norms and standards of professional practice  b. Planning to ensure cohesion by being aware of difficult situations the team is facing  c. Assuring feedback and visibility of front-line managers and medical leaders |
| **Watch video extract 2 of the vignette** | |
| **Shared mental models**  **(Stability, Team functioning, Team cohesion)** | 1. This extract describes a form of identification with teamwork. How does this apply here?   a. Acknowledges complexity  b. Clarifies roles and stability  c. Shared vision of the goals of the person living with or beyond cancer |
| **Watch video extract 3 of the vignette** | |
| **Shared mental models**  **(Communication, Coordination, Team practice environment)** | 1. What mechanisms are in place to facilitate teamwork?   a. Clinical meeting  b. Intervention plans  c. Oncology passport and communication tools  d. Being together and sharing thoughts / Needing to be alone |
| **Watch video extract 4 of the vignette** | |
| **Shared mental models**  **(Sense of coherence)** | 1. How do people living with and beyond cancer affect your capacity for resilience?   a. What does that mean to you (feeling of competence vs. learning through practice)?  b. How does the feeling of coherence manifest: what makes sense?  c. How does the feeling of coherence manifest: what does not make sense? |
| **Shared mental models**  **(Perception of adversity, Quality of life at work, Team resilience at work)** | 1. Can you describe a particularly positive experience where the team recovered from a difficult situation? 2. Can you describe a less positive experience where the team had trouble recovering from a difficult situation? |
| **Conclusion** | 1. If I had just one message to deliver from you to promote team resilience at work in oncology, what would it be and to whom would I bring it? |
| **Acknowledgment** | We are at the end of this group discussion. Thank you very much for your time and for sharing your knowledge and experience! |

1. **Group interview guide for component C^2^: Photovoice**

| **Participating site ID** |  |
| --- | --- |
| **Group ID code** |  |
| **Date** |  |
| **Start time** |  |
| **End time** |  |
| **Interviewing team members and roles** | Interviewer:  Co-interviewer:  Note-taker: |
| **Introduction and ground rules** | |
| **Interviewer’s introduction** | Thank you for taking the time and making yourselves available for this group discussion and for the project. |
| **Confidentiality and anonymity** | I would like to remind you that any information you reveal here will remain confidential. Under no circumstances your names or occupations will be transmitted when the results of the study will be presented or published. If names are mentioned during the discussion, they will be anonymized. Only members of the research team will have access to the information for analysis purposes, and they are all committed to respecting the confidentiality of information.  You can withdraw from the study at any time if you want to, without any consequences. Do you have any questions?  Please read and sign the consent form. |
| **Audio recording** | This interview will be audio recorded. The audio recording will be transcribed to respect anonymity.  Please feel free to ask me to stop the recording at any moment for specific questions if needed.  Also, if you wish to remove some content, you can ask me during or immediately after the interview. |
| **Introduction** | I will ask you some questions about the different aspects of resilience at work in oncology teams. What I am interested in is your perspective, experience and perceptions on the subject, so feel free to add anything you think is relevant: there is no good or bad answer or point of view. |
| **Photovoice preparation** | Please select one or two pictures that best represent team resilience at work. |
| **Themes** | **Questions for collecting qualitative data (SHOWED)^1^** |
| **Shared mental models (Team resilience at work)** | 1. What do you **S**ee on the picture you selected? |
| **Shared mental models (Adverse situations, Actions/mechanisms)** | 1. What do you think is actually **H**appening in this picture? |
| **Shared mental models (Team resilience at work)** | 1. How does the story in the picture relate to **O**ur team's resilience at work? |
| **Shared mental models (Team, organizational and healthcare system context)** | 1. **W**hy do things happen like this?    1. Team level    2. Organizational level    3. Healthcare system level |
| **Shared mental models (Actions/mechanisms)** | 1. How might this picture **E**nlighten us on actions that support team resilience at work? |
| **Conclusion** | 1. What can we **D**o to make it happen? |
| **Acknowledgment** | We are at the end of this group discussion. Thank you very much for your time and for sharing your knowledge and experience! |

SHOWED approach adapted from Photovoice Steering Committee (2007) Manual and Resource Kit: Photovoice. Hamilton, Ontario: National Association of County Health Officials; p. 38, <https://www.naccho.org/uploads/downloadable-resources/Programs/Public-Health-Infrastructure/Photovoice-Manual.pdf>

1. **Group interview guide for component C^4^: Uptake and feedback**

| **Participating site ID code** |  |
| --- | --- |
| **Group ID code** |  |
| **Date** |  |
| **Start time** |  |
| **End time** |  |
| **Interviewing team members and roles** | Interviewer:  Co-interviewer:  Note-taker: |
| **Introduction and ground rules** | |
| **Interviewer’s introduction** | Thank you for taking the time and making yourselves available for this group discussion and for the project. |
| **Confidentiality and anonymity** | I would like to remind you that any information you reveal here will remain confidential. Under no circumstances your names or occupations will be transmitted when the results of the study will be presented or published. If names are mentioned during the discussion, they will be anonymized. Only members of the research team will have access to the information for analysis purposes, and they are all committed to respecting the confidentiality of information.  You can withdraw from the study at any time if you want to, without any consequences. Do you have any questions?  Please read and sign the consent form. |
| **Audio recording** | This interview will be audio recorded. The audio recording will be transcribed to respect anonymity.  Please feel free to ask me to stop the recording at any moment for specific questions if needed.  Also, if you wish to remove some content, you can ask me during or immediately after the interview. |
| **Introduction** | I will ask you some questions about the different aspects of resilience at work in oncology teams. What I am interested in is your perspective, experience and perceptions on the subject, so feel free to add anything you think is relevant: there is no good or bad answer or point of view. |
| **Themes** | **Questions for collecting qualitative data** |
| **Interpretation of results and prioritization of actions** | 1. Following presentation of intervention results:   a. What surprises you?  b. What actions would help the most to minimize, manage and mend from adversity? |
| **Intervention context – Individual level** | 1. What motivated you/would motivate you to be involved:    1. in the intervention    2. in future plans? |
| **Intervention context – Organizational level** | 1. Was the intervention compatible with local needs and practices? |
| **Intervention outcomes** | 1. Did the intervention have the anticipated effects (or not)? Any other effects? Please explain. |
| **Participation outcomes** | 1. Describe one positive thing that came out of your participation? 2. Describe one less positive thing that came out of your participation? |
| **Learnings** | 1. Describe one thing that could be improved for scaling interventions to other oncology teams? |
| **Conclusion** | 1. What is a key message that comes out of your participation? |
| **Acknowledgment** | We are at the end of this group discussion. Thank you so much for your time and generosity in sharing your knowledge and experience! |

1. **Individual interview guide for key informants**

| **Participating site ID code** |  |
| --- | --- |
| **Participant ID code** |  |
| **Date** |  |
| **Start time** |  |
| **End time** |  |
| **Interviewer** |  |
| **Introduction and ground rules** | |
| **Interviewer’s introduction** | Thank you for taking the time and making yourself available for this group discussion and for the project. |
| **Confidentiality and anonymity** | I would like to remind you that any information you reveal to me will remain confidential. If names are mentioned during the discussion, they will be anonymized. Under no circumstances your name or occupation will be transmitted when the results of the study will be presented or published. Only members of the research team will have access to the information for analysis purposes, and they are all committed to respecting the confidentiality of information.  You can withdraw from the study at any time if you want to, without any consequences. Do you have any questions?  Please read and sign the consent form. |
| **Audio recording** | This interview will be audio recorded with your permission. The audio recording will be transcribed so as to respect anonymity.  Please feel free to ask me to stop the recording at any moment for specific questions if needed.  Also, if you wish to remove some content, you can ask me during or immediately after the interview. |
| **Introduction** | I will ask you some questions about the different aspects of resilience at work in oncology teams. What I am interested in is your perspective, experience and perceptions on the subject, so feel free to add anything you think is relevant: there is no good or bad answer or point of view. |
| **Themes** | **Questions for collecting qualitative data** |
| **Interviewee’s role** | - What is your profession/responsibility? - Tell me about your experience in your work? - What is your role in regard with oncology teams? |
| **Mental models (Resilience at work)** | - When you hear 'Team resilience at work in oncology', what does it mean to you? - What importance is given to team resilience in your workplace? Please specify. |
| **Mental models (Context, adverse situations)** | - How would you describe the environment, i.e. the clinical and organizational context, in which the oncology team operates?   1. Organizational changes?   2. Practice changes?   3. Work environment?   4. Workplace politics? |
| **Oncology team functioning, team cohesion** | - Tell me about the oncology team :   1. How do people identify as part of the team?   2. How do members support each other? (i.e. back-up)   3. What other means of supporting each other are evident?   4. How does trust develop within the team?   5. What helps team functioning? (e.g., meetings, social activities, communication practices)   6. What would improve team functioning? |
| **Leadership (Resources and expertise)** | - How do leaders support team resilience? - What could they do better or more of? |
| **Organizational level initiatives supporting team resilience capacity** | - Describe examples of strategies/actions put in place to:  1. Minimize the effects of difficult situations 2. Manage difficult situations 3. Mend (recover and learn)  - What are the challenges involved in adopting strategies to support resilience in the oncology team? |
| **Health system level initiatives supporting team resilience capacity** | - What indicators are used to measure team well-being and resilience in oncology? - What links do you see between team resilience at work in oncology teams and the National Cancer Program? - What concrete actions related to governance could be proposed or put in place to support oncology team resilience? |
| **Conclusion** | - If I had just one message to deliver from you to promote team resilience at work in oncology, what would it be and to whom would I bring it? - Is there anything else you consider important to this study? |
| **Acknowledgment** | - We are at the end of this interview. Thank you very much for your time and for sharing your knowledge and experience! |
