# Supplementary material for: Building resilience in oncology teams: Protocol for a realist evaluation of multiple cases
Source: PLoS One. 2022 May 12;17(5):e0268393. doi: 10.1371/journal.pone.0268393 (PMC9098052; doi:10.1371/journal.pone.0268393)
Supplement: S2 File — (DOCX) [file pone.0268393.s002.docx]

**S2 File. Elements evaluated in the study**

The following table presents elements included in qualitative and/or quantitative analysis. Sections S2A (Context), S2B (Target mechanisms of the intervention) and S2C (Outcomes) describe elements incorporated in the conceptual framework, while section S2D presents an element regarding the appreciation of group. French versions of questionnaires will be used in the study. “Descriptors” provides short operational definitions of each element. “Data sources” refers to data collected to pursue the aims of the study. Number of items and reliability coefficients (Cronbach alphas) are provided. “Types of data” refers to qualitative or quantitative data, as well as the scales for the quantification strategy of qualitative data [1].

| **S2A. CONTEXT** | | | |
| --- | --- | --- | --- |
| ***Team level*** | | | |
| **Elements** | **Descriptors** | **Data sources** | **Types of data** |
| Shared mental models [2] | Understanding of the work environment and of each other’s tasks and responsibilities, allowing members to act appropriately and efficiently as a team | Discourse | - Qualitative data - Quantification: Present=1 / Absent=0 |
|  |  | Documentation   - Written role description | - Quantification: Present=1 / Absent=0 |
|  |  | Documentation   - Written referral criteria to team members | - Quantification: Present=1 / Absent=0 |
|  |  | Documentation   - Regular formal team meetings | - Quantification: Present=1 / Absent=0 / Occasionally=0.5 |
| Resources and expertise | Identified medical leader | Discourse | - Qualitative data - Quantification: Present=1 / Absent=0 |
|  | Visibility of front-line manager | Discourse | - Qualitative data - Quantification: Present=1 / Absent=0 |
|  | Continuing education | Discourse | - Qualitative data - Quantification: Present=1 / Absent=0 |
|  |  | Documentation   - Written formal program | - Quantification: Present=1 / Absent=0 |
| Stability | Short-term absenteeism | Documentation   - % of absences ≤ 3 consecutive days | - % from 0 to 100 |
|  | Long-term sick leave | Documentation   - % of absences > 3 consecutive days | - % from 0 to 100 |
|  | Unfilled positions | Documentation   - Number of unfilled positions by type of profession | - Continuous |
|  | Turnover rate | Documentation   - % of team members who left the oncology team definitively | - % from 0 to 100 |
| Characteristics | Team size [3] | Documentation   - Number of professionals in the team | - Categorical   < 8 = Small team  ≥ 8 = Large team |
|  | Team composition [3] | Documentation   - Types of professionals in the team | - Categorical |
|  |  | Documentation   - % of different professions represented by team members | - % from 0 to 100 for each type of profession |
|  |  | Documentation  • Average age: years | - Continuous |
|  |  | Documentation   - Average professional experience: years | - Continuous |
|  |  | Documentation   - Average professional experience in the team: years | - Continuous |
| ***Individual level*** | | | |
| **Elements** | **Descriptors** | **Data Sources** | **Types of data** |
| Quality of life at work [4] | Perceived quality of life at work | Discourse | - Qualitative data - Quantification: Present=1 / Absent=0 / Occasionally=0.5 |
|  |  | Questionnaire   - Numerical scale: visual thermometer | - 0-100:   < 25 = Requires intervention  25-50 = Requires improvement  > 50 = Good |
| Perception of adversity | Description of work-related difficult situations | Discourse | - Qualitative data - Quantification: Present=1 / Absent=0 / Occasionally=0.5 |
|  | Perceived impact of adversity related toCOVID-19 | Questionnaire   - Numerical scale: visual analog scale | - 0-100 |
| Sense of coherence at work  (Work-SoC Questionnaire [5]) | Making sense at work; based on to what extent the work situation is comprehensible, manageable and meaningful | Questionnaire   - 9 items; α=0.83 - Semantic differential scale; 7 levels | - Score |
| Personal accomplishment/burnout [6] | Feelings of being competent and of successfully achieving work | Questionnaire   - 8 items; α=0.72 - Likert-type scale; 7 levels with scores from 0 to 6 | - Score:   < 33 = Low  34-39 = Moderate  ≥ 40 = High |
| Characteristics | Age: years | Questionnaire   - Number | - Continuous |
|  | Gender: self-identification | Questionnaire   - Response choices: woman, man, non-binary, prefers not to answer, prefers being identified as … | - Categorical |
|  | Gender-related roles: roles that may influence work and intervention components; responsibilities relative to partner/spouse  Questionnaire: Labour Force Gender Index (LFGI) [7] | Questionnaire   - 4 items, α=not available - 3 to 5 response choices | - Score:   Higher scores = more traditionally feminine  Lower scores = more traditionally masculine |
|  | Education level: highest level completed | Questionnaire   - Response choices: secondary, college or CEGEP*, university | - Categorical |
|  | Profession: type of profession | Questionnaire   - Response choices: oncologist, nurse, social worker, psychologist, clerical personal, physiotherapist, pharmacist, spiritual care provider, other | - Categorical |
|  | Professional experience: years | Questionnaire   - 5 response choices | - Categorical |
|  | Professional experience in the oncology team: years | Questionnaire   - 5 response choices | - Categorical |
|  | Workhours: average hours worked per week | Questionnaire   - Number | - Continuous |
|  | Professional role: type of role | Questionnaire   - Response choices: clinician, front-line manager, administrative, other | - Categorical |
| ***Organizational level*** | | | |
| **Elements** | **Descriptors** | **Data sources** | **Types of data** |
| Support initiatives | Type and description of local support initiatives or programs accessible to oncology team members | Discourse | - Qualitative data - Quantification: Present=1 / Absent=0 |
|  |  | Documentation | - Qualitative data - Quantification: Present=1 / Absent=0 |
| Resources | Resources inventory accessible to and used by oncology team members | Discourse | - Qualitative data - Quantification: Present=1 / Absent=0 / Occasionally=0.5 |
|  |  | Documentation | - Qualitative data - Quantification: Present=1 / Absent=0 / Occasionally=0.5 |
| Feedback on quality of care | Type of quality of care indicators | Discourse | - Qualitative data - Quantification: Present=1 / Absent=0 |
|  |  | Documentation | - Qualitative data - Quantification: Present=1 / Absent=0 |
|  | Frequency and mechanisms of feedback | Discourse | - Qualitative data - Quantification: Present=1 / Absent=0 / Occasionally=0.5 |
|  |  | Documentation | - Qualitative data - Quantification: Present=1 / Absent=0 / Occasionally=0.5 |
| Access to workforce | Local workforce shortage | Discourse | - Qualitative data - Quantification: Present=1 / Absent=0 |
|  |  | Documentation | - Qualitative data - Quantification: Present=1 / Absent=0 |
|  |  | Documentation   - % of workforce shortage | - % from 0-100 |
| Characteristics | Mandate: Type of organizational mandate related to oncology care | Documentation   - Local, regional, supra regional | - Categorical |
|  | Academic affiliation: Type of affiliation | Documentation   - University hospital, community hospital | - Categorical |
|  | Size of deserved population | Documentation   - Number | - Continuous |
|  | Geographic location: Type of location | Documentation   - Mega urban, urban, semi-rural, rural | - Categorical |
|  | Staff turnover rate | Documentation   - % of employees who left the organization definitively | - % from 0-100 |
| ***Healthcare system level*** | | | |
| **Elements** | **Descriptors** | **Data sources** | **Types of data** |
| National Cancer Plan | Program and main priorities: integration of national cancer plan elements and workforce challenges | Discourse | - Qualitative data - Quantification: Present=1 / Absent=0 / Occasionally=0.5 |
|  |  | Documentation | - Qualitative data - Quantification: Present=1 / Absent=0 / Occasionally=0.5 |
|  | Guidelines and protocols in place during our intervention: influence on workload | Discourse | - Qualitative data - Quantification: Present=1 / Absent=0 / Occasionally=0.5 |
|  |  | Documentation | - Qualitative data - Quantification: Present=1 / Absent=0 / Occasionally=0.5 |
|  | Governance model: modes of coordination, collaborative regiments, network-based approaches | Discourse | - Qualitative data - Quantification: Present=1 / Absent=0 / Occasionally=0.5 |
|  |  | Documentation | - Qualitative data - Quantification: Present=1 / Absent=0 / Occasionally=0.5 |
|  | Structure: committees and modes of communication, type of leadership | Discourse | - Qualitative data - Quantification: Present=1 / Absent=0 / Occasionally=0.5 |
|  |  | Documentation | - Qualitative data - Quantification: Present=1 / Absent=0 / Occasionally=0.5 |
| Resources | Publicly funded system: cancer specialist coverage, linkages with community organizations | Discourse | - Qualitative data - Quantification: Present=1 / Absent=0 / Occasionally=0.5 |
|  |  | Documentation | - Qualitative data - Quantification: Present=1 / Absent=0 / Occasionally=0.5 |
| COVID-19 guidelines | Guidelines to face pandemic adversity | Discourse | - Qualitative data - Quantification: Present=1 / Absent=0 / Occasionally=0.5 |
|  |  | Documentation | - Qualitative data - Quantification: Present=1 / Absent=0 / Occasionally=0.5 |
| **S2B. TARGET MECHANISMS** | | | |
| ***Mechanisms activating resilience*** | | | |
| **Elements** | **Descriptors** | **Data sources** | **Types of data** |
| Minimizing [8] | Actions taken before an adverse situation occurs or as soon as it appears, to support team resilience at work capacity | Discourse  Assess, anticipate and plan; assess and understand readiness; detect warning signs; get ready to handle stressors | - Qualitative data - Quantification: Present=1 / Absent=0 / Occasionally=0.5 |
| Managing [8] | Actions taken during an adverse situation, to support team resilience at work capacity | Discourse  Assess difficulties rapidly and accurately; address sources of chronic stress; provide support; keep processes going; ask for advice | - Qualitative data - Quantification: Present=1 / Absent=0 / Occasionally=0.5 |
| Mending [8] | Actions taken after an adverse situation, to support team resilience at work capacity | Discourse  Reassess the situation; learn from what happened (e.g. debriefing); deal with concerns; adapt and get ready for further adverse situations | - Qualitative data - Quantification: Present=1 / Absent=0 / Occasionally=0.5 |
| ***Mechanisms activating teamwork*** | | | |
| **Elements** | **Descriptors** | **Data sources** | **Types of data** |
| Communication (Relational Coordination [9-11]) | Communicating frequently, timely, accurately and with a focus on problem-solving | Discourse | - Qualitative data - Quantification: Present=1 / Absent=0 / Occasionally=0.5 |
|  |  | Questionnaire   - - 4 items out of 7 (7 items; α=0.86) - Likert-type scale; 5 levels | - Score |
| Coordination (Relational Coordination [9-11]) | Having shared goals, shared knowledge and mutual respect | Discourse | - Qualitative data - Quantification: Present=1 / Absent=0 / Occasionally=0.5 |
|  |  | Questionnaire   - - 3 items out of 7 (7 items; α=0.86) - Likert-type scale; 5 levels | - Score |
| Team practice environment (Practice Environment Checklist mini-PEC [12]) | Sharing ideas, environment suitable to accomplishment, availability of the information needed to perform, efforts to understand problems, ability to act according to the team vision | Discourse | - Qualitative data - Quantification: Present=1 / Absent=0 / Occasionally=0.5 |
|  |  | Questionnaire   - 5 items, α=0.82 - Likert-type scale; 4 levels | - Score |
| **S2C. OUTCOMES** | | | |
| ***Outcome: Team resilience at work*** | | | |
| **Elements** | **Descriptors** | **Data sources** | **Types of data** |
| Resourcefulness  (R@W Team sub-scale [13]) | Leveraging team strengths and resources. Focusing on continuous improvement. Building team processes that are effective and priority-oriented | Questionnaire   - 10 items; α=0.93 - Likert-type scale; 7 levels | - Sub-scale score |
| Robustness  (R@W Team sub-scale [13]) | Sharing common purpose, meaning and goals. Ability to adapt to change and be proactive about problem solving | Questionnaire   - 8 items; α=0.85 - Likert-type scale; 7 levels | - Sub-scale score |
| Perseverance  (R@W Team sub-scale [13]) | Focusing on solutions not problems. Being tenacious when facing obstacles | Questionnaire   - 3 items; α=0.83 - Likert-type scale; 7 levels | - Sub-scale score |
| Self-care  (R@W Team sub-scale [13]) | Promoting stress management practices and identifying signs of overload. Encouraging work/life balance | Questionnaire   - 7 items; α=0.87 - Likert-type scale; 7 levels | - Sub-scale score |
| Capability  (R@W Team sub-scale [13]) | Asking for feedback on performance. Capitalizing on successful practices. Developing skills and knowledge | Questionnaire   - 7 items; α=0.89 - Likert-type scale; 7 levels | - Sub-scale score |
| Connectedness  (R@W Team sub-scale [13]) | Cooperating, supporting each other and encouraging a sense of belonging to the team | Questionnaire   - 2 items; α=0.81 - Likert-type scale; 7 levels | - Sub-scale score |
| Alignment  (R@W Team sub-scale [13]) | Aligning towards objectives. Being optimistic. Acknowledging progress and success | Questionnaire   - 5 items; α=0.88 - Likert-type scale; 7 levels | - Sub-scale score |
| Team Resilience at Work  (R@W Team (full) scale [13]) | Dynamic process and work team members' ability to deal effectively with adversity situations | Questionnaire   - 42 items; α=0.95 - Likert-type scale; 7 levels | - Total score |
| ***Outcome: Teamwork*** | | | |
| **Elements** | **Descriptors** | **Data sources** | **Types of data** |
| Team functioning (Team functioning scale [14]) | Interpersonal support: Respecting each other, sharing work-related information or ideas, resolving differences, and helping each other | Questionnaire   - 12 items; α=0.84 - Likert-type scale; 5 levels | • Score |
| Team cohesion (Interdisciplinary Team Performance [15]) | Identifying with the oncology team and its common goals | Questionnaire   - 7 items; α=0,86 - Likert-type scale; 5 levels | - Score |
| Health related quality of life  (CORE-6D [16] | CORE-6D: Frequency of occurrences related to emotional health (feeling lonely, panicked, humiliated, suicidal, able to accomplish things) and physical health (pain or other physical issues) | Questionnaire   - 6 items, R^2^=0.99 - Lickert-type scale; 5 levels | - Score |
| Health-related quality of life  (SF-6Dv2 [17]) | SF-6Dv2: Ability to undertake physical, daily and social activities. Experience of physical pain. Time spent feeling depressed or very nervous; feeling worn out | Questionnaire   - 6 items, α=0.84 - 4 to 6 response levels | - Score |
| Cost-effectiveness | Cost of resources for the intervention, including human resources and consumable goods | Documentation   - Canadian dollars ($) | - Continuous |
|  | Economic evaluation of cost-effectiveness ratio | Calculation of incremental cost-effectiveness ratio (ICER) from CORE-6D and SF-6Dv2 outcomes | - ICER ratio |
|  | Demonstration of quality-adjusted life years (QALY) of oncology team members at different points during the intervention, to determine quantity and quality of life | QALY calculation | - QALY scores |
| **S2D. GROUP DISCUSSION APPRECIATION** | | | |
| **Element** | **Descriptor** | **Data source** | **Type of data** |
| Group discussion evaluation [18] | Evaluation of productivity, efficiency and environment during group discussions around intervention components 1, 2 and 4 | Questionnaire   - Numerical scale: visual analog scale | - 0-10 |

* College/CEGEP: In Québec, colleges and CEGEP are post-secondary institutions that provide education in preparation for university (2 years), as well as vocational and technical education programs (3 years).

**S2 File References**

1. Caracelli VJ, Greene JC. Data analysis strategies for mixed-method evaluation designs. Educ Eval Policy Anal. 1993;15(2):195-207. doi: 10.3102/01623737015002195.

2. Gucciardi DF, Crane M, Ntoumanis N, Parker SK, Thøgersen-Ntoumani C, Ducker KJ, et al. The emergence of team resilience: a multilevel conceptual model of facilitating factors. J Occup Organ Psychol. 2018;91(4):729-68. doi: 10.1111/joop.12237.

3. Tremblay D, Roberge D, Touati N, Maunsell E, Berbiche D. Effects of interdisciplinary teamwork on patient-reported experience of cancer care. BMC Health Serv Res. 2017;17(1):218. doi: 10.1186/s12913-017-2166-7.

4. Dupuis G, De Grandi M. L'inventaire systémique de qualité de vie au travail (ISQVT): un outil de mesure opérationnel pour aller au-delà des RPS. In: Les Cahiers de l'Actif, editor. Management d'équipes et Qualité de vie au travail. 484-487: Actif Information; 2016. p. 215-29.

5. Vogt K, Jenny GJ, Bauer GF. Comprehensibility, manageability and meaningfulness at work: construct validity of a scale measuring work-related sense of coherence. SA J Ind Psychol. 2013;39(1):1-8. doi: 10.4102/sajip.v39i1.1111.

6. Maslach C, Jackson S. Maslach Burnout Inventory: Manual. 2nd ed. Palo Alto, CA: Consulting Psychologists Press; 1986. 34 p.

7. Smith PM, Koehoorn M. Measuring gender when you don’t have a gender measure: constructing a gender index using survey data. Int J Equity Health. 2016;15(1):82. doi: 10.1186/s12939-016-0370-4.

8. Alliger GM, Cerasoli CP, Tannenbaum SI, Vessey WB. Team resilience: how teams flourish under pressure. Organ Dyn. 2015;44(3):176-84. doi: 10.1016/j.orgdyn.2015.05.003.

9. Gittell JH. Coordinating mechanisms in care provider groups: relational coordination as a mediator and input uncertainty as a moderator of performance effects. Manage Sci. 2002;48(11):1408-26. doi: 10.1287/mnsc.48.11.1408.268.

10. Gittell JH, Beswick J, Goldmann D, Wallack SS. Teamwork methods for accountable care: Relational coordination and TeamSTEPPS®. Health Care Manage R. 2015;40(2):116-25. doi: 10.1097/HMR.0000000000000021.

11. Gittell JH, Seidner R, Wimbush J. A relational model of how high-performance work systems work. Organ Sci. 2010;21(2):490-506. doi: 10.1287/orsc.1090.0446.

12. Lurie SJ, Schultz SH, Lamanna G. Assessing teamwork: a reliable five-question survey. Fam Med. 2011;43(10):731-4. PubMed PMID: 22076717.

13. McEwen K, Boyd CM. A measure of team resilience: developing the resilience at work team scale. J Occup Environ Med. 2018;60(3):258-72. doi: 10.1097/JOM.0000000000001223.

14. Rousseau V, Aubé C, Savoie A. Le fonctionnement interne des équipes de travail: conception et mesure [Internal functioning of work teams: conception and measurement]. Can J Behav Sci. 2006;38(2):120-35. doi: 10.1037/cjbs2006002.

15. Temkin-Greener H, Diane G, Dana M, Kunitz SJ. Measuring interdisciplinary team performance in a long-term care setting. Med Care. 2004;42(5):472-81. doi: 10.1097/01.mlr.0000124306.28397.e2.

16. Mavranezouli I, Brazier JE, Rowen D, Barkham M. Estimating a preference-based index from the Clinical Outcomes in Routine Evaluation-Outcome Measure (CORE-OM): valuation of CORE-6D. Med Decis Making 2013;33(3):381-95. Epub 2012/11/25. doi: 10.1177/0272989X12464431.

17. Mulhern BJ, Bansback N, Norman R, Brazier J. Valuing the SF-6Dv2 classification system in the United Kingdom using a discrete-choice experiment with duration. Med Care. 2020;58(6):566-73. Epub 2020/03/30. doi: 10.1097/mlr.0000000000001324.

18. St-Arnaud Y. Les petits groupes: Participation et animation. 3rd ed. Montreal, QC: Gaétan Morin: Chenelière; 2008. 192 p.
